# A scoping review of mentorship in Graduate Medical Education: a proposed conceptual framework

**DOI:** 10.3389/fmed.2025.1616148

**Published:** 2025-08-18

**Authors:** Dima Abdelmannan, Rasha Buhumaid, Hira Salman, Wail A. Abdulrahman Hasan Ba Madhaf, Hafidh Mohammad Khamis AlRajaby, Nabil Zary, Shaista Salman Guraya

**Affiliations:** ^1^Graduate Medical Education, Mohammed Bin Rashid University of Medicine and Health Sciences, Dubai Health, Dubai, United Arab Emirates; ^2^Emergency Medicine, Dubai Health, Dubai, United Arab Emirates; ^3^Institute of Learning, Mohammed Bin Rashid University of Medicine and Health Sciences, Dubai Health, Dubai, United Arab Emirates

**Keywords:** Graduate Medical Education, mentorship, mentoring outcomes, mentor-mentee relationship, theoretical frameworks, scoping review framework

## Abstract

**Introduction:**

Mentorship is increasingly recognized as a foundational stone within Graduate Medical Education (GME), contributing to clinical competency, scholarly engagement, professional identity formation, and psychological well-being. Despite its growing recognition, mentorship in GME remains inconsistently structured, under-theorized, and variably evaluated. This conceptual and structural ambiguity hampers the ability to design, compare, and scale mentorship efforts meaningfully across settings. This scoping review aimed to systematically explore the structure, theoretical foundations, evaluation approaches, and reported outcomes of mentorship programs in GME, and to develop a conceptual framework to guide the design of context-sensitive, outcome-aligned mentorship interventions.

**Methods:**

The scoping review followed Arksey and O’Malley’s five-stage methodology and the findings were reported according to PRISMA-ScR guidelines. A comprehensive search of PubMed, Scopus, CINAHL, and Embase was conducted in January 2025, covering studies published between 2015 and 2025. Eligibility was defined using the Population–Concept–Context framework. Data were extracted using a structured template and synthesized thematically.

**Results:**

A total of 94 studies were included. Mentorship programs varied widely in structure, with formal, informal, peer, and near-peer models observed. Only 27 studies reported use of theoretical frameworks, and evaluation approaches were often limited to non-validated tools and descriptive outcomes. Four main analytical clusters emerged: program structure, theoretical/conceptual frameworks, evaluation approaches, and reported outcomes. Outcomes commonly reported included career development, academic productivity, clinical competency, leadership, well-being, and professional growth. However, the main highlight was a lack of theoretical underpinnings, standardized outcome measurement and mentor training. Cultural responsiveness and equity were rarely considered in mentorship programs.

**Conclusion:**

This scoping review highlights the need for mentorship programs in GME to be more systematically designed, theory-informed, and rigorously evaluated. Key gaps include the underutilization of conceptual models, the lack of validated evaluation tools, and insufficient attention to mentor training and equity considerations. Building on the findings of this scoping review, we propose a conceptual framework that aligns mentorship models with learner level, skill focus, and mentoring format across psychological and sociological domains. This framework is intended to guide the development of robust, context-sensitive, and theory-informed mentorship programs with measurable outcomes, ultimately fostering sustainable mentorship cultures that enhance learner development and improve healthcare practice in Graduate Medical Education (GME).

## Introduction

Mentorship is increasingly recognized as a foundational stone within Graduate Medical Education (GME), contributing to clinical competency, scholarly engagement, professional identity formation, and psychological well-being (1, 2). At its core, mentorship refers to a sustained, developmental relationship in which a more experienced professional (the mentor) provides guidance, support, and feedback to a less experienced individual (the mentee), to promote personal, professional, and academic growth (3). Increasingly, mentorship is recognized not only as a means of individual development (4) but also as a strategic mechanism for promoting workforce stability, faculty and trainee retention, engagement, and institutional commitment (5). It plays a critical role in addressing systemic challenges such as burnout, workforce attrition, and leadership gaps in healthcare systems (1, 3).

Studies from both medical education and organizational contexts have shown that effective mentorship reduces attrition, improves morale (2), and enhances alignment between individual goals and institutional missions. Yet, academic health systems continue to struggle with integrating education, research, and clinical care under a unified vision challenges that are compounded by the multifaceted demands of mentorship, particularly within traditionally siloed structures (6, 7). In such complex environments, mentorship can serve as a high-impact intervention that drives learning (8) and development on multiple levels. Individually, it enhances skill acquisition (9, 10), academic engagement (11), professional identity formation, and career clarity (1, 12–14). At the organizational level, mentorship supports leadership development, institutional alignment (9), scholarly productivity (11, 15), and retention (12). Societally, mentorship contributes to improved patient care and public health outcomes, reinforcing the ethical and professional obligations academic institutions have toward their communities (13).

Despite its growing recognition, mentorship in GME remains inconsistently structured, under-theorized, and variably evaluated (1, 3). Programs differ significantly in how they are implemented ranging from informal, *ad hoc* pairings to formal, structured initiatives. Many fail to clearly define their objectives, whether focused on technical skill acquisition, non-technical competencies, or career development leading to unchecked “performance gaps” (8). Key variables such as the mentee’s stage of training, the mentor’s preparation, and expected outcomes are often overlooked. Additionally, mentorship is still largely confined to traditional one-on-one faculty-trainee models, with limited integration of alternative formats like peer, group, or inverse mentorship (16). Evaluation strategies also lack standardization, frequently relying on informal feedback or satisfaction surveys rather than validated instruments or theory-informed assessments (10, 17). This conceptual and structural ambiguity hampers the ability to design, compare, and scale mentorship efforts in a meaningful way across settings.

While several prior reviews have explored mentorship in medical education, most fall short of offering a comprehensive, GME-specific analysis (18). Many are limited to select specialities or focus narrowly on outcomes such as publication productivity or satisfaction, without addressing mentorship as a developmental, relational, and contextually embedded process (2, 4, 9–11, 14, 15, 18–21). Very few provide institutions with actionable frameworks for designing or evaluating mentorship programs in diverse clinical environments. This scoping review, conducted and reported following the PRISMA-Scr (Preferred Reporting Items for Systematic Reviews and Meta-Analyses extension for Scoping Reviews) guidelines (22, 23) addresses these limitations by offering a systematic, pan-disciplinary synthesis of mentorship practices across GME. It maps the structure and characteristics of both formal and informal mentorship programs; examines the theoretical frameworks underpinning these interventions; and evaluates reported outcomes, including competency development, career progression, leadership capacity, and professional identity formation. This scoping review aims to support academic health systems in designing outcome-driven, context-sensitive mentorship programs that align with institutional missions and evolving workforce needs.

## Methods

### Study design

As per PRISMA-ScR guidelines (22, 23), this scoping review aims to map the existing literature on mentorship in GME by systematically exploring the characteristics and structure of mentorship programs, including formal and informal models, the theoretical frameworks underpinning mentorship interventions, and the reported outcomes such as professional development, competency acquisition, career progression, and professional identity formation. Additionally, it seeks to identify gaps in the current literature to inform future research and guide the development of evidence-based mentorship programs in GME. The scoping review adhered to the five-stage methodological framework (24) proposed by Arksey and O’Malley (25) that is well-suited for broad, interdisciplinary topics that require exploratory mapping of key concepts, evidence gaps, and practical applications. We did not apply the optional sixth stage—consultation with external stakeholders—since this review was conducted to first inform internal institutional design efforts.

### Stage 1: identifying the research questions

This scoping review was guided by one primary and several supporting research questions aimed at providing a comprehensive understanding of mentorship in Graduate Medical Education (GME).

*Primary question*: What are the structural features, conceptual frameworks, and reported outcomes of mentorship programs in Graduate Medical Education?

*Secondary questions*:

- How are mentorship models structured across different disciplines, training stages, and institutions?
- What theoretical frameworks (if any) are used to design or evaluate these programs?
- What types of outcomes-technical, non-technical, career-related, or psychosocial are most frequently reported for both mentors and mentees?
- What evaluation methods are applied, and what gaps persist in literature?

### Stage 2: identification of relevant studies

The search strategy was developed with academic librarians and applied across four databases: PubMed, Scopus, CINAHL, and Embase. Searches were conducted in January 2025 and limited to articles published between 2015 and 2025. A combination of keywords and MeSH terms was used, including: *“mentorship,” “mentors,” “graduate medical education,” “residency,” “fellowship,” “medical education,” “training,”* and related terms (see the search strategy in Box 1). For a detailed description of search strategy, please refer to Appendix 1.

BOX 1 **Entailing the primary search strategy developed using PubMed**

Pubmed 19th Feb 2025 = 1,697

Search: **Graduate Medical Education AND Mentorship** Filters: **from 2015–2025**

((“education, medical, graduate”[MeSH Terms] OR (“education”[All Fields] AND “medical”[All Fields] AND “graduate”[All Fields]) OR “graduate medical education”[All Fields] OR (“graduate”[All Fields] AND “medical”[All Fields] AND “education”[All Fields])) AND (“mentors”[MeSH Terms] OR “mentors”[All Fields] OR “mentorship”[All Fields] OR “mentorships”[All Fields])) AND (2015:2025[pdat])

**Translations**

**Graduate Medical Education:** “education, medical, graduate”[MeSH Terms] OR (“education”[All Fields] AND “medical”[All Fields] AND “graduate”[All Fields]) OR “graduate medical education”[All Fields] OR (“graduate”[All Fields] AND “medical”[All Fields] AND “education”[All Fields])

**Mentorship:** “mentors”[MeSH Terms] OR “mentors”[All Fields] OR “mentorship”[All Fields] OR “mentorships”[All Fields]

No language or study design filters were initially applied to ensure comprehensive coverage. Reference lists of included studies were hand-screened for additional citations.

### Stage 3: selection of studies

All records retrieved from the database searches were imported into Covidence for deduplication, screening, and review management. Screening was conducted independently by three reviewers DA, RB and HS, who assessed the relevance of each study based on predefined inclusion and exclusion criteria. Articles that appeared eligible were subjected to a full-text review, during which 148 publications were independently assessed. Disagreements regarding inclusion were resolved through group consensus meetings to ensure methodological rigor and consistency.

Eligibility criteria were developed using the Population–Concept–Context (PCC) framework recommended for scoping reviews (24). Studies were included if they involved postgraduate medical trainees such as residents, fellows, or interns; explored structured mentorship programs, theoretical frameworks, or mentorship-related outcomes; and were situated within GME contexts such as hospitals or academic medical centers. Empirical studies including qualitative, quantitative, mixed-methods designs, and review articles with analytical insights were eligible. Studies were excluded if they focused solely on undergraduate students or non-medical professionals, described mentorship only as a minor component, centered on purely technical skills, or were conducted outside clinical or postgraduate training environments. Only studies published between 2015 and 2025 were considered, aligning with the scope of contemporary GME practice. The complete inclusion and exclusion criteria are summarized below in Table 1.

**Table 1:** Detailed overview of inclusion exclusion criteria.

Criterion	Included	Excluded
Population	Postgraduate medical trainees (residents, fellows, interns)	Undergraduate medical students, non-medical professions (unless interprofessional mentorship)
Concept	Structured mentorship programs, theoretical frameworks, mentorship outcomes	Mentorship as a minor component, purely technical skill training
Context	GME settings (hospitals, academic medical centers)	Non-clinical mentorship programs
Study design	Empirical studies (qualitative, quantitative, mixed-methods, reviews with insights)	Systematic reviews, meta-analyses, commentaries without empirical data
Time frame	2015–2025	Studies before the cut-off year (if any)

### Stage 4: data charting

DA and HS independently examined each article that qualified for full-text review. Data were charted using a structured template adapted from Covidence’s v2.0 scoping review matrix and guided by the PRISMA-ScR reporting guidelines (26). The charting framework captured descriptive variables such as author(s), year of publication, country, and medical specialty. In addition, we recorded the career stage of participants (resident, fellow, or faculty), the type of mentorship described (formal, informal, peer, near-peer, or faculty-led), and structural program elements including duration, frequency of meetings, and mentor-mentee pairing strategies. To support conceptual mapping, we also documented the presence of any theoretical or conceptual frameworks, the evaluation approaches used (including instruments, study design, and metrics), and the reported outcomes for both mentors and mentees. DA and HS initially charted the data independently, after which all authors reviewed the consolidated dataset. Discrepancies were resolved through iterative discussion until consensus was reached. Final agreement on overarching concepts and key constructs informed the subsequent stages of descriptive-analytical analysis.

### Stage 5: data synthesis

The primary research question focused on identifying the structural features, conceptual frameworks, and reported outcomes of mentorship programs within Graduate Medical Education (GME). To support this inquiry, this scoping review examined how mentorship models are structured across disciplines, stages of training, and institutional contexts. It also explored the presence and application of theoretical or conceptual frameworks guiding program design or evaluation. Additionally, outcomes commonly reported for both mentors and mentees spanning technical, non-technical, career-related, and psychosocial domains were cataloged. Finally, this scoping review assessed the evaluation methods used and highlighted persistent gaps in the literature. SSG conducted the analysis, drafted the results and the initial and final draft of the manuscript. SSG first conducted a descriptive analysis to summarize key study characteristics, including country of origin, medical speciality, and career stage of participants. This was followed by a narrative synthesis to explore the complexity of mentorship practices. Studies were then mapped onto a conceptual continuum, ranging from psychology-oriented models focused on skill acquisition and structured feedback to sociology-driven models emphasizing identity formation and integration within professional cultures. Four major analytical clusters emerged from this synthesis: (1) mentorship program structures, (2) theoretical and conceptual frameworks, (3) evaluation approaches, and (4) reported outcomes. These clusters provided a scaffold for deeper inferential interpretation, culminating in the development of a conceptual framework proposed later in this scoping review.

Throughout this process, attention was given to recurring gaps, including the limited use of validated evaluation tools, insufficient mentor training, and the absence of theory-informed program design. These analytical insights directly informed the construction of a context-sensitive, outcome-aligned framework for mentorship interventions in GME. A constructivist worldview (27) influenced SSG’s approach to data synthesis, which was iteratively acknowledged throughout the interpretive nature of qualitative data analysis. To ensure transparency and rigor, all team members, DA, RB, HS, WB, HR, NZ and SSG, remained actively engaged through continuous cross-checking, clarification of interpretations, and iterative feedback. All co-authors reviewed and endorsed the final synthesis, emergent framework and the write up.

## Results

**Figure 1:**
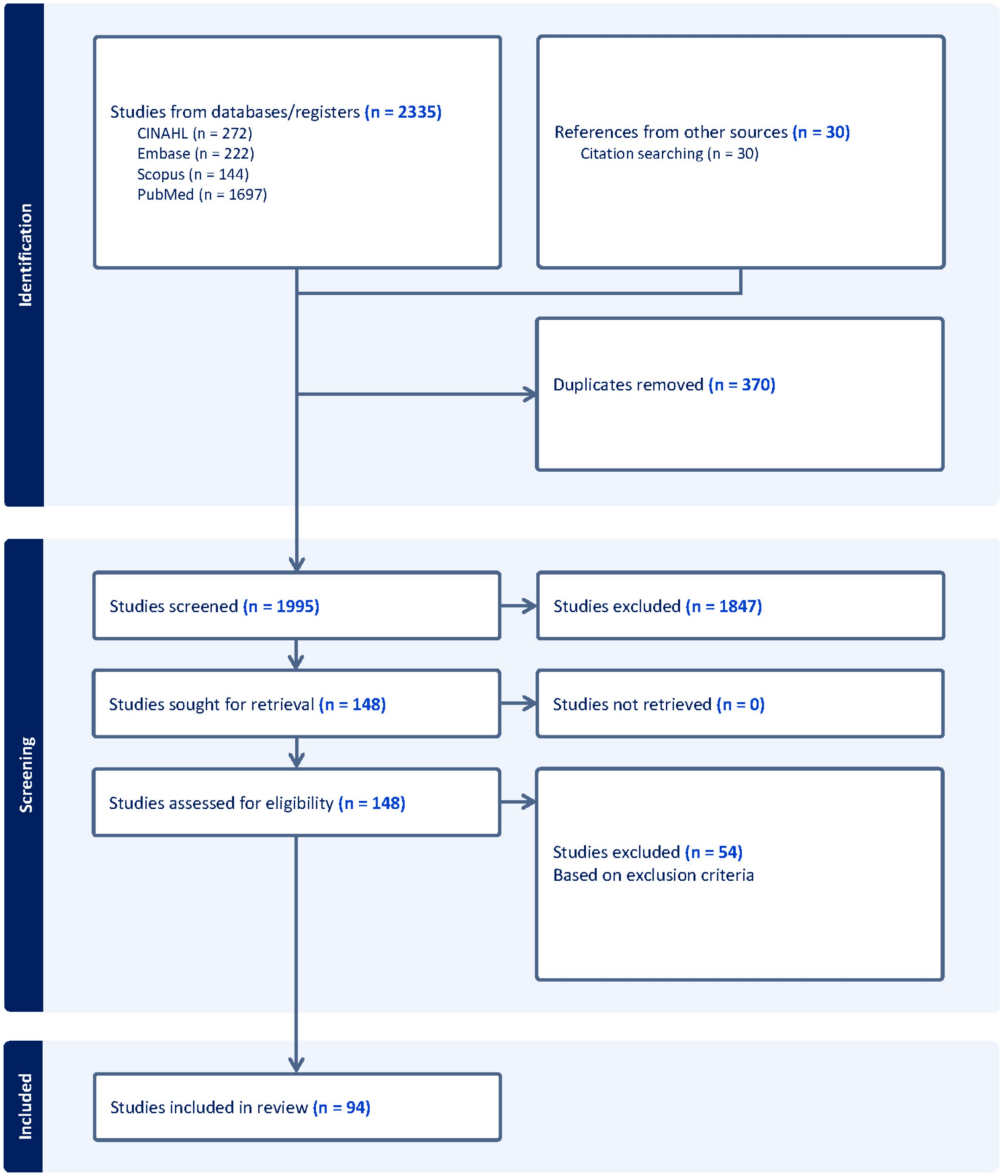
PRISMA flow diagram outlines the study selection process, starting with 2,335 studies and narrowing to 94 studies after applying inclusion and exclusion criteria.

Our search for peer-reviewed articles in January 2025 yielded 2,335 articles from the four databases, with an additional 30 articles identified through citation searching. 370 duplicated records were excluded. Then, 1995 unique studies were screened for relevance based on their titles and abstracts. A total of 148 studies were selected for full-text screening, and which ultimately resulted in 94 studies that met the inclusion criteria (Figure 1).

### Study characteristics

This scoping review analyzed 94 (2–4, 6, 9–21, 28–104) studies published predominantly between 2017 and 2023. Out of 94 studies, fifty-eight were conducted in the United States (2–4, 10–14, 18–21, 30, 35, 36, 39, 40, 42, 44–50, 52, 57, 59, 60, 62–66, 69–71, 74–76, 78–80, 82–84, 86, 89–92, 94, 97–99, 103, 104) followed by 15 studies from Canada (6, 16, 17, 34, 38, 43, 53, 56, 58, 61, 73, 85, 93, 96, 101), with smaller contributions from countries such as Qatar (31, 41, 68), Pakistan (9, 77, 100), and the United Kingdom (29). The mentorship interventions studied spanned from academic medical centers to varied size hospital settings (9, 12, 13, 15, 29, 42, 47, 48, 50, 51, 55, 60, 62, 64, 70, 72, 75, 81, 87, 97, 100, 102). The populations studied included residents, fellows, and faculty members. Study designs varied, with a mix of quantitative (surveys), qualitative (interviews, focus groups), and pre/post comparative evaluations. However, the majority of studies were descriptive in nature and lacked longitudinal follow-up. In terms of structured mentor training only 26.6% of studies described details of any form such as workshops, toolkits, or orientation sessions. In contrast, 73.4% of studies did not report offering mentor preparation, suggesting that while mentorship programs are commonly implemented, the deliberate development of mentor capacity remains an under-addressed aspect of program design. Table 2 outlines the characteristics of included studies.

**Table 2:** Characteristics of the 94 included studies.

Characteristic	No. (%) of studies
Publication year	
2016 or earlier	17 (18.1%)
2017–2023	75 (79.8%)
Region	
United States	58 (61.7%)
Canada	15 (16.0%)
Qatar	3 (3.2%)
Pakistan	3 (3.2%)
Others (UK, Japan, Brazil, etc.)	15 (16.0%)
Disciplines	
Surgery	15 (16.0%)
Anesthesiology	5 (5.3%)
Radiology	5 (5.3%)
Internal Medicine	4 (4.3%)
Pediatrics	3 (3.2%)
Emergency	3 (3.2%)
Psychiatry	2 (2.1%)
Career stage	
Residents	69 (73.4%)
Faculty	31 (33.0%)
Fellows	4 (4.3%)
Study design	
Descriptive (narrative, program reports survey-based)	24 (25.5%)
(cross-sectional, perceptions)	37 (39.4%)
Pre–post evaluation	8 (8.5%)
Quasi-experimental	3 (3.2%)
Longitudinal cohort	2 (2.1%)
Literature review	5 (5.3%)
Mixed-methods	12 (12.8%)
Randomized controlled trials (RCTs)	0 (0.0%)

### Mentorship program structures

Mentorship programs demonstrated wide variation in structure and implementation. Of the 94 studies, 80 implemented formal mentorship programs (2, 4, 9, 11–15, 17–19, 21, 28–30, 32, 33, 35–38, 40–42, 45, 47, 48, 50–70, 72–83, 85, 88–92, 94–96, 98–103) characterized by assigned mentor-mentee pairings, defined objectives, and institutional oversight. Several studies described structured mentorship programs with formal meeting schedules, clearly defined goals, and evaluation mechanisms. Notably, Gusic et al. (18) detailed strategies for designing an effective structured mentorship program; while, Patel et al. (30) implemented a formal, structured evaluation of faculty mentorship of the formal structured mentoring program. The faculty mentors were assigned to junior trainees, whereas the senior trainees self-selected their mentors: required contract signing and biannual mentor-mentee meetings with recorded interactions. Cohee et al. (35) incorporated mentor-guided self-directed learning milestones and compared the quality of formal mentoring relationships with preexisting informal mentoring relationships for internal medicine residents. Structured meetings and workshop attendance were mandatory, and a yearlong mentor-mentee “chemistry” played a role in increasing the proportion of residents staying with mentor from 50 to 96%. However, Amen et al. (40) integrated formal mentorship into palliative care education for surgical residents and fellows. A structured curriculum was introduced, featuring small-group discussions, role-playing exercises, and direct faculty feedback and its impact was measured on a more systems-level in the form of ICU quality metrices and documentation quality instead of mentor-mentee perceptions. Additional examples include Bhatia (47) in 2016 which emphasized a long-term goal-oriented mentoring; designed and evaluated a 4-year mentorship program that integrated academic development, clinical teaching, and ACGME-competency-based assessment for emergency medicine residents. Residents were assigned an advisor in their first year and then selected a mentor for the remainder of training. Program components included quarterly development meetings, simulation sessions, direct clinical observations, assistance with academic presentations, and career development discussions. Chan et al. (51) in 2021 with defined mentor-mentee pairing to enhance research productivity. Mentors assisted residents in research design, literature review, data collection, manuscript writing, and presentation skills. Two steps evaluation showed 100% of the residents presented at grand roubds and 52% published a manuscript in peer-reviewed journal. These structured designs offer models for consistency, accountability, and scalability across mentorship initiatives. In contrast, several studies included both formal and informal models, highlighting the hybrid nature of mentorship structures in graduate medical education which developed organically and offered relational flexibility but lacked accountability structures. One study from Qatar (51) featured structured research alignment and periodic meetings, albeit in a more laissez-faire manner, highlighting the need for faculty training to improve effectiveness.

Faculty mentors were featured in majority of the studies, while peer mentorship appeared in 24 studies (2, 3, 9, 10, 14–16, 19, 39, 42, 49, 52, 56, 62, 67, 77–79, 82–84, 90, 93, 96) and near-peer models in 10 studies (2, 9, 10, 14, 19, 52, 62, 67, 82, 93). Group mentorship (15, 16, 28, 30, 52, 59, 60, 64, 70, 71, 91), virtual formats (15, 30, 42, 64), and longitudinal models (29, 57) were also represented, although their representation was inconsistent. Mentorship was particularly prevalent in certain medical disciplines. Surgery accounted for the highest number of discipline-specific studies (9, 16, 17, 20, 21, 28–32, 37, 49, 50, 65, 66, 70, 71, 83, 86, 87, 90, 96, 97, 99, 102–104), followed by anaesthesiology (38, 58, 59, 61, 74, 85, 88, 93), pediatrics (5, 31, 41, 55, 64, 72, 75), and radiology (2, 52, 62, 73, 80). However, over half of the studies did not specify a medical specialty in the title or abstract, suggesting either a generalist approach to mentorship or limited reporting of disciplinary context.

**Figure 2:**
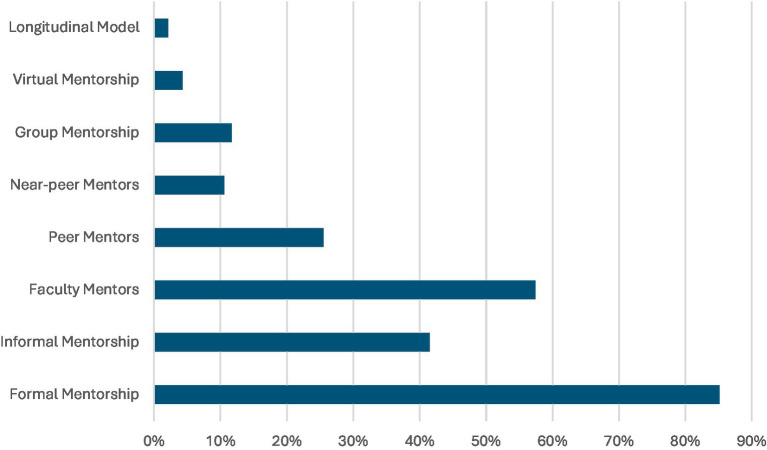
Proportion of the 94 included studies (primarily published between 2017 and 2023) that reported on elements of mentorship programs in Graduate Medical Education (GME).

Despite inconsistencies in reporting, a few studies described clearly defined mentor-mentee pairing mechanisms. For example, mechanism of structured or assigned mentor-mentee pairings were reported in very few (16, 18, 28, 30, 31). These mechanisms included formal matching processes (28, 30), one-to-one assignments (16, 31), or deliberate alignment based on interest or specialty (18). The inconsistent documentation of mentorship duration, meeting frequency, and mentor-mentee pairing mechanisms underscores the need for greater programmatic clarity and standardization. Among those that did report it, Khair, Abdulrahman (31) outlined a structured monthly meeting format; Amen, Berndtson (40) described regular feedback cycles within its pairing system; and surgical training mentorship program (9) emphasized scheduled, structured interactions between mentors and mentees; and Flurie, Hylton Gravatt (60) included a clearly defined frequency as part of its mentorship model. Additionally, mentor training (35, 39, 45, 59, 74, 75, 92) discussed included workshops or preparatory guidance for mentors. A few studies also specified mentorship duration, describing a one-year longitudinal model (41), while a USA based study reported a semester-based (92) culturally responsive mentorship cycle. However, such specification was uncommon across the broader dataset (Figure 2).

### Theoretical and conceptual frameworks

The use of theory across mentorship interventions was uneven. Of the 94 studies included, 27 explicitly articulated a theoretical or conceptual framework (3, 10, 16, 18, 29–32, 34, 36–38, 40–42, 44–46, 48–50, 52, 53, 58, 62, 63, 70, 71). These frameworks varied in disciplinary origin, conceptual depth, and the extent to which they were integrated across study design, delivery, and evaluation. Among the most commonly cited frameworks were the halstedian apprenticeship model (29, 53) used to describe traditional hierarchical clinical teaching; Socratic Inquiry (46), which emphasized dialogical and reflective mentor-mentee engagement; and adult learning theory (36) which guided adult learner-centered mentorship strategies. Experiential learning theory (40, 41), social learning theory (52), sociocultural theory (10), and communities of practice (62) also appeared across studies to frame learning processes.

Other frameworks, such as self-determination theory, transformative learning, and professional identity formation were conceptually mentioned but not systematically applied within any of the studies. Only a few studies operationalized theory throughout their mentorship model. For example, Aho (36) explicitly used Knowles’ andragogy to guide mentor strategies, while Amen (40) structured its program around Kolb’s learning cycle. Cheng (52) applied Bandura’s model to address psychosocial and academic support needs for behavioral modeling. Many studies referenced theory vaguely or *post hoc* without aligning their methods or outcomes to a theoretical base. A majority lacked a transparent conceptual model, often conflating program design with educational strategy. This undermines the rigor and transferability of findings.

The theoretical frameworks employed can be broadly categorized into two types: psychology-driven theories and socially situated learning models with a very few overlapping both disciplines (Table 3).

**Table 3:** Enumeration of various theories used and their categorization into disciplinary origins.

Psychology driven theories	Sociology driven theories
Experiential learning theories Self-determination theory Transformative learning theory Role modeling theory Knowles adult learning theory Plato–Aristotle relationship Freud and Jung philosophy Professional identity formation Self-efficacy theory	Halstedian apprenticeship approach Socratic inquiry Applied complexity theory Social network theory Social learning theory Mosaic mentorship model Vygosky’s zone of proximal development Social support theory Critical race theory Theory of Practice Theory of sponsorship
Peer-mentorship models Krama and Higgins framework Managing up theory	

Psychology-driven theories focused on individual learning, reflection, and motivation such as adult learning theory (36), Experiential Learning Theory (40, 41), social learning theory (52), and conceptually, self-determination theory and transformative learning. In contrast, socially situated theories positioned learning within communities and relationships such as sociocultural theory (10), communities of practice (62), halstedian apprenticeship (29, 53), and socratic inquiry (46). This categorization shows that while mentorship draws on a rich theoretical landscape, few studies attempted to combine or contrast psychological and social learning perspectives to enhance program design. A more substantial commitment to theory-informed approaches is necessary to elevate mentorship from an operational tool to a field of scholarly inquiry.

### Evaluation approaches

Evaluation methods varied, mostly employing surveys or questionnaires. Rest of the studies did not report any clearly defined evaluation method, highlighting a significant gap in systematic program assessment. One-fourth of the analyzed studies (13, 15, 34, 56, 58, 67, 68, 73, 75, 77, 78, 83, 86, 88, 91, 92, 99–101) used qualitative methods such as interviews and focus groups to explore mentorship experiences. While 19 conducted pre/post or comparative evaluations to assess outcomes over time (4, 14, 15, 18, 19, 34, 39, 40, 50, 54–56, 66, 68, 71, 76, 79, 80, 93). Most evaluations were descriptive; few utilized mixed-methods or longitudinal approaches. While effectiveness was frequently claimed in almost all of the studies, methodological rigor was often lacking. Only one study by Zhang, Isaac (17) explicitly reported using a validated inventory in its evaluation specifically, the Mentor Effectiveness Scale (MES), a previously published and psychometrically validated tool for assessing mentor qualities and impact. Additionally, Caine, Schwartzman (49) applied the Context, Input, Process, Product (CIPP) model to structure a comprehensive evaluation of the mentorship program. The CIPP model allowed for evaluation across multiple stages of implementation, linking program rationale, design inputs, execution processes, and measured outcomes in a unified framework. These examples reflect a broader reliance across the literature on self-developed or non-standardized tools. Notably, five studies reported limited or no effectiveness. Wadhwa, Nagy (4) revealed misalignment between mentor and mentee expectations, resulting in perceived ineffectiveness. Smeds et al. (70) found no statistically significant impact on long-term career progression. However, Korbitz et al. (83) reported minimal change in burnout or resilience indicators, attributing this to short intervention duration. These findings underscore the importance of rigorous program design, mentor engagement, and robust evaluation strategies. These findings emphasize the importance of thorough program design, mentor engagement, and robust evaluation strategies. Measures of impact focused predominantly on subjective gains such as perceived increases in confidence, self-efficacy, clarity around career decisions, feelings of professional support, motivation, and overall satisfaction with the mentoring relationship, rather than on externally assessed or performance-based outcomes, reflecting a broader reliance on self-developed or non-standardized tools.

### Reported outcomes

Mentorship was consistently associated with a wide range of positive outcomes across the 94 studies, as shown in Box 2.

BOX 2 **Studies reporting the identified outcomes**

**Career development** was interpreted as specialty selection, academic advancement, and long-term planning (2–4, 6, 9, 11–21, 28, 29, 32, 37–39, 41, 45–47, 51–56, 58, 59, 61–64, 68, 72–75, 78–80, 85–92, 94–97, 99–102).

**Academic productivity** was interpreted as increased research involvement, publications, presentations, and scholarly collaborations (6, 9–11, 13, 15, 17, 21, 37, 41, 43–47, 51, 60, 62, 66, 69–73, 75–77, 80, 85, 90, 92, 94, 97–99, 102, 104).

**Well-being** was interpreted as **i**mproved emotional support, psychological safety, and reduced burnout (2, 17, 19–21, 29, 37–39, 45, 55, 56, 63, 67, 69, 77, 78, 80, 83, 86, 88–91, 94, 97).

**Clinical competency** was documented as growth in clinical knowledge and capacity, including technical skills (9) (e.g., Study 22) and broader clinical development (55, 94) with limited reference to diagnostic acumen or interprofessional communication (9, 11, 20, 47, 51, 53, 54, 61, 65, 66, 94, 99, 101, 103).

**Professional growth** was recorded as enhanced confidence, autonomy, identity formation, and reflective practice (4, 9–11, 15, 17, 18, 20, 28, 41, 47, 56, 58, 60, 73, 76–78, 86, 92, 95, 96, 101).

**Leadership skills** were recorded as development of decision-making, advocacy, and role modeling capacities (9, 11, 12, 20, 21, 52, 62, 65, 67, 77, 78, 82, 87, 94, 97).

These findings affirm mentorship’s multifaceted contributions to learner development and institutional scholarship. However, most outcomes were measured through self-reported surveys assessing perceived improvements in career direction (9, 29, 57), research involvement (3, 18, 72), emotional support (37, 60), and clinical preparedness (43, 48, 55). Academic productivity was typically tracked via the number of publications, conference presentations, or research projects reported by mentees (36, 50, 75). Professional growth was gauged through reflections, self-efficacy scores, or qualitative narratives (6, 10, 46). A few studies measured burnout and well-being using inventories such as the Maslach Burnout Inventory (4, 57, 83), while leadership skills were often inferred from self-perceived role expansion, advocacy, or involvement in teaching and mentoring others (32, 52, 85). Overall, there was a lack of standardized outcome measures and limited use of objective performance indicators.

## Discussion

**Figure 3:**
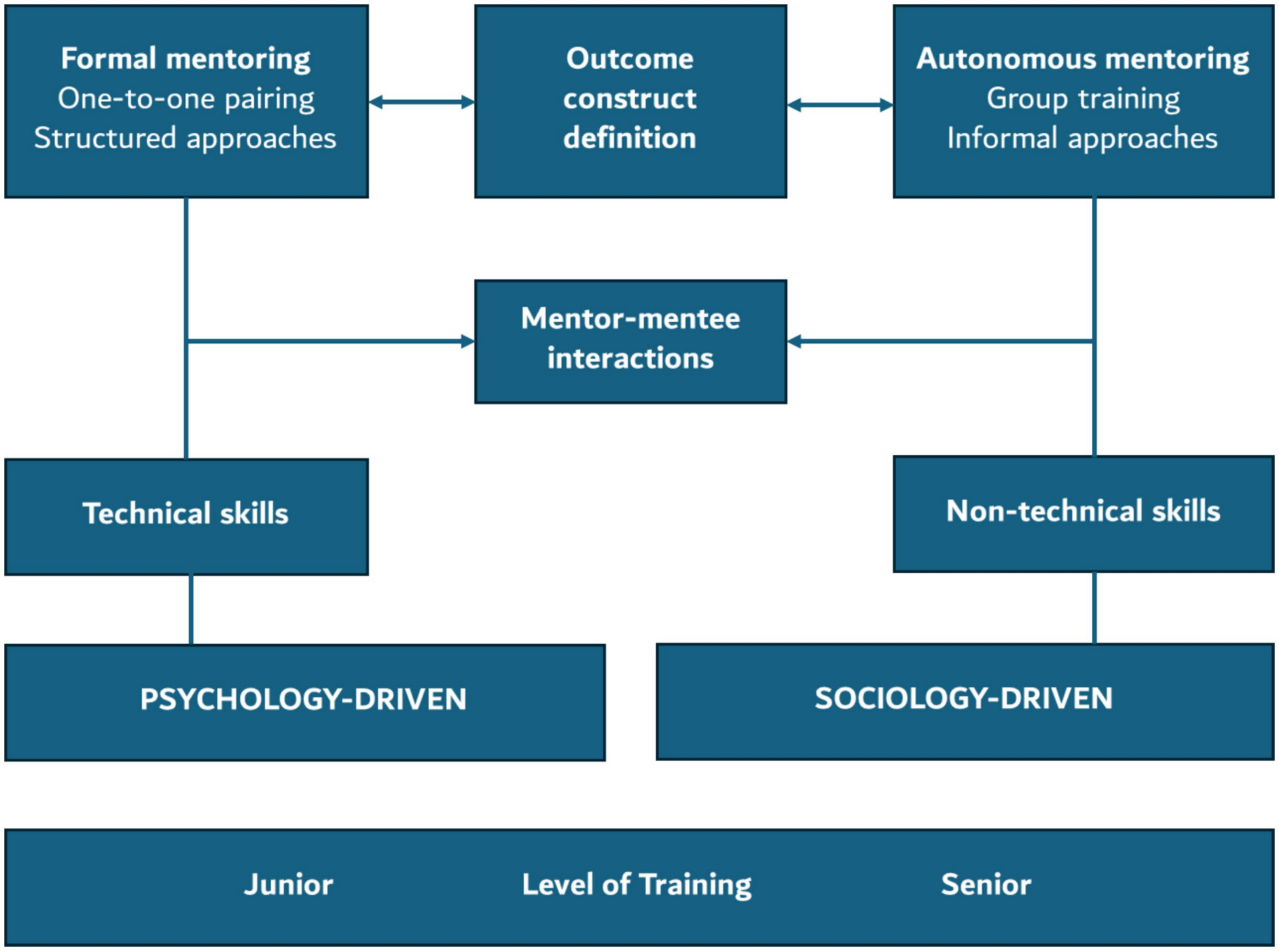
Conceptual framework illustrating key components of mentorship design in GME, spanning psychological to sociological approaches, mapped against learner stage, skill focus, and mentor–mentee interactions.

Mentorship is increasingly recognized as an essential component of Graduate Medical Education (GME), valued for its multifaceted contributions to clinical competency, academic productivity, professional identity formation, and overall career advancement. Despite the widespread advocacy for mentorship in policy and practice, this scoping review of 94 studies highlights substantial heterogeneity in how mentorship is conceptualized, implemented, and evaluated across settings. The field lacks consensus on key components of effective mentorship programs, and there remains a significant gap in rigorous, theory-informed, and outcome-aligned models. This scoping review provides a foundational synthesis of mentorship interventions over the past two decades and offers a starting point for creating shared understanding around program design and effectiveness. Several themes emerged that warrant further exploration and practical attention. These include the scarcity of tailored interventions, the underuse of formal mentorship training particularly for mentees; a lack of consistent and validated outcome measures; and the need for more robust experimental designs, including randomized controlled trials (RCTs). In response, we propose a conceptual framework (Figure 3) informed by this synthesis that integrates psychological and sociological paradigms, offering a developmental pathway for mentorship models aligned with learners’ evolving needs and institutional objectives.

Our proposed conceptual model for mentorship captures the key components of effective intervention design. This model is visually represented in Figure 3 and incorporates mentor–mentee interaction mechanisms and the definition of outcome constructs as core elements. This framework that integrates psychological and sociological paradigms to guide the design of mentorship interventions in GME. Structured across three key dimensions: learner level, skill focus, and mentorship format, with mentor–mentee interactions and outcome construct definition serving as central anchors. On the psychology-driven end, mentorship is formal, structured, and focused on technical skill development ideal for junior learners such as early-stage residents. These interactions typically involve one-to-one, mentor-led formats emphasizing task mastery, clinical reasoning, and procedural proficiency. Disciplines such as surgery, anesthesiology, and radiology frequently adopt this model to foster early clinical competence. As learners advance, the framework transitions toward sociology-driven mentorship, which is more autonomous, relational, and suited for senior residents and faculty. Here, the emphasis shifts to non-technical skills like leadership, professionalism, empathy, and identity formation. Informal and peer-based mentoring formats, such as those in internal medicine, psychiatry, and pediatrics, support reflective learning, psychosocial development, and role transition. Mentor–mentee interactions evolve from guided instruction to peer dialog and community-based support. The framework also identifies core mentorship outcomes, including academic productivity, well-being, identity integration, and leadership, as essential to track and evaluate. By adopting this framework, institutions and researchers can better align mentorship interventions with learner needs and educational goals. In the following sections, we critically examine the findings of this scoping review through the lens of this conceptual model. Each framework dimension, learner level, mentorship format, skill focus, interaction type, outcome construct, mentor development, and evaluation design is an organizing structure for interpreting how current mentorship practices align with educational theory and learner needs.

### Theoretical grounding of programs

The findings of this scoping review reveal a diverse yet fragmented theoretical landscape in mentorship literature within health professions education. While early career mentorship programs tended to align with psychology-driven models emphasizing skill acquisition and structured learning these were largely applied descriptively rather than operationalized. Similarly, sociology-driven approaches for senior learners such as peer mentoring, sponsorship, and mentorship networks were again inconsistently grounded in theory. Across the dataset, the incorporation of theory ranged from superficial mentions (theory-dropping) to mere framing; none demonstrated meaningful theoretical conversation or application (105). However, one study (19) stood out by applying “Managing Up” theory from business management to reframe mentorship through a mentee-centered lens. This approach empowered surgical trainees to take ownership of their mentorship relationships and provided a clear conceptual pathway that informed both program structure and expected outcomes. Such theoretically anchored designs not only improve conceptual clarity but also help address implementation gaps, an outcome rarely achieved in theory-absent models. These inconsistencies underscore that designing complex mentorship interventions is inherently challenging. Adopting a design-based approach (106) can help navigate this complexity, allowing for more iterative, theory-informed program development that aligns with both learner needs and institutional goals.

### Skills development across training stages

While technical and non-technical skills are frequently cited outcomes of mentorship, only a few studies in this scoping review explicitly delineated between these domains or explored how they relate to learner stage and disciplinary context. Technical skills refer to discipline-specific competencies, such as procedural proficiency, diagnostic accuracy, and hands-on clinical techniques. These were more commonly emphasized in junior learners, particularly within procedure-intensive disciplines like surgery, anesthesiology, and radiology. For instance, Bassett et al. (44) in the surgical mentorship literature synthesis highlighted the role of mentorship in developing surgical dexterity, academic productivity, and performance benchmarking, especially for early-career residents. In contrast, non-technical skills encompass communication, empathy, leadership, professionalism, and identity integration. These were more prominent in studies involving peer and faculty mentorship in internal medicine, pediatrics, and psychiatry, where psychosocial support and relational development were foregrounded. Khair et al. (31) examined mentorship among pediatric residents using a one-to-one faculty model. While the study did report elements of interpersonal growth and reflective support, its primary findings centered on mentee satisfaction and the value of structured guidance and feedback. Fournier (16) explored a flexible, peer-based mentoring model that emphasized psychological safety, emotional resilience, and community building among residents. Similarly, another study (39) evaluated a mentorship intervention in a Medicine-Pediatrics residency program, which integrated faculty and peer mentorship and resulted in improvements in leadership confidence, empathy, and reflective capacity. However, another interesting outcome of operating comfort levels were also assed by Smeds et al. (70).

These studies collectively suggest that technical skills are prioritized in the early stages of training, aligning with structured, psychology-driven mentorship models, whereas non-technical skills such as identity formation, leadership, and emotional resilience emerge as focal points in later stages, typically supported by informal, sociologically grounded approaches. Despite their importance, many programs did not explicitly define or assess these skill domains, presenting an opportunity for future mentorship models to more intentionally align skill development with learner progression and contextual needs.

### Mentorship outcomes from the mentee and the mentor perspective

Our scoping review affirms that mentorship in GME yields a wide range of benefits for both mentees and mentors, with notable patterns that can inform future program design. Mentees most commonly reported gains in career development, academic productivity, and clinical skill enhancement. For instance, mentees documented improved research output and academic advancement (44), while some linked mentorship to increased career satisfaction and professional engagement (12). Emotional well-being and resilience were also consistently cited, particularly in structured or near-peer programs using assessed using faculty feedback and resident reflections (53) and a notable mitigation of burnout (79) stress reduction, especially when mentees were proactive (19) and enhance belonging, especially for residents from underrepresented backgrounds (83). Programs that enabled mentee choice in mentor pairing or offered structured matching processes showed higher satisfaction and stronger engagement (14).

For mentors, benefits were less frequently reported but equally meaningful. Mentors cited professional growth, personal satisfaction, and a sense of giving back as major drivers of engagement (37, 68). Faculty mentors (76) reported increased leadership skills and communication abilities after participating in mentorship programs. In structured settings, mentorship also contributed to academic productivity and institutional recognition (56). Several studies noted that mentor engagement improves when faculty receive training, are matched based on shared interests, or when mentorship is formally recognized as part of academic promotion pathways (70, 82). Nonetheless, recurrent challenges were reported, including lack of protected time, unclear mentoring roles and limited institutional support. These findings suggest that sustainable mentorship models require investment in mentor development, structured feedback mechanisms, and systems-level acknowledgment of mentorship as a core academic contribution.

However, there is a glaring absence of three critical dimensions that could significantly enhance the scope and impact of mentorship programs. First, inverse mentorship where junior faculty or trainees mentor seniors in areas like digital fluency can offer opportunities for bidirectional learning and mutual growth. Second, clearer differentiation between mentorship and supervision can reduce role conflicts, fostering safer, more developmental spaces, particularly when mentors come from outside a trainee’s specialty (11). Third, faculty themselves need structured mentorship to sustain engagement, promote institutional alignment, and reinforce mentorship as a shared organizational value. Programs should consider integrating multi-role mentorship models tailored to different learner stages and goals, ensuring alignment with both career-focused and psychosocial support objectives (10, 39, 46).

### Mentorship models and roles of a mentor

This scoping review reveals a dominant reliance on formal mentorship models which are typically structured by institutions with predefined mentor-mentee assignments and scheduled interactions. While such models are efficient for oversight and consistency, they may limit relational flexibility and responsiveness to individual mentee needs, mainly when mentor-mentee matching is not based on mutual interest or compatibility. For example, Khan et al. (34) highlighted that residents often did not consider assigned mentors as true mentors, suggesting a lack of perceived alignment and relational engagement. Despite their widespread use, formal programs often do not account for the nuanced, developmental transitions trainees experience. Faculty mentorship was reported in majority of studies usually within hierarchical dyads *“train at the feet of master”* (21). Patel et al. (30) described a formal model led by faculty mentors, while Khair et al. (31) included structured faculty-paired mentorship focusing on interpersonal growth and reflective support.

A significant portion of studies described informal mentorship, which emerged organically through collegial interactions. These relationships often provided greater psychosocial support, flexibility, and authenticity, but their lack of structure could lead to unequal access with some trainees benefiting more simply due to personality, initiative, or social capital (2). Similarly Bortnick (46)emphasized that self-initiated mentorship relationships tended to result in better alignment and satisfaction. While no study explicitly concluded that informal mentorship is categorically superior, several findings collectively imply that such models can offer more responsive and supportive environments, especially when formal mentorship fails to meet relational or psychosocial needs. Interestingly, self-initiated and hybrid models were compared in only one study (46).

Peer mentorship and near-peer mentorship emerged as impactful models especially in fostering psychological safety, shared experiences, and community support among junior residents. Fournier et al. (16) described peer-based psychosocial support; Blitz (39) mentioned a hybrid faculty and peer model that improved leadership confidence; and Caine (49) used a novel speed-dating format to form peer matches. While some incorporated senior residents mentoring juniors using structured engagement (52) few other proposed a hybrid faculty-peer model but did not operationalize it (38). These underexplored models indicate a gap in designing mentorship systems that reflect evolving needs, where multiple mentors can address technical skills, emotional well-being, and career advancement concurrently.

Few studies explicitly labeled mentors as “coaches, sponsors, or connectors,” (81) “educators and role models” (46) though these functions were often implied in programs that emphasized leadership development and network-building. These findings highlight the importance of role clarity, mentor training, and structural support in shaping effective mentorship ecosystems. Programs should consider integrating multi role mentorship models tailored to different learner stages and goals, and evaluate not just presence but quality and role diversity within mentorship relationships.

### Mentor training and capacity building

Despite the increasing emphasis on formal mentorship programs in GME, our scoping review found that only a limited number of studies (2, 11, 12, 42, 52) described any form of structured mentor training. Even among these, the nature and depth of training varied substantially. Toklu et al. (11) introduced feedback calibration tools and a “win-win contract” approach, while some (67) offered short peer mentor preparation workshops emphasizing psychological safety and shared experience. Aylor (42) et al. incorporated a structured mentorship toolkit featuring worksheets and templates to guide expectation setting and progress tracking. Each of these models contributed in some way to mentor preparedness, with reported improvements in communication quality, engagement, and alignment of expectations. In another study by Faloye et al. (59), mentioned structured approach worth noting involved faculty mentors who voluntarily enrolled and underwent Mentor Competency Assessment (MCA) training at both the outset and conclusion of the program. Mentees (residents) ranked their preferred mentors from this trained pool, and matches were finalized based on these preferences, allowing no more than two mentees per mentor. This structured, feedback-driven process ensured alignment of expectations, enhanced mentor accountability, and promoted sustained mentor-mentee engagement. Another innovative program design was grounded in a composite of evidence-informed mentorship training models (92). Together, these frameworks provided a multidimensional foundation to structure mentor development and evaluation across domains such as cultural responsiveness, communication, self-efficacy, and long-term planning.

However, the impact of these training strategies was overwhelmingly assessed through subjective indicators, such as mentor-reported confidence or mentee satisfaction, rather than through objective evaluation metrics. Additionally, many mentorship programs that encountered issues such as low mentor engagement (29), mismatched expectations (10, 38, 47, 56, 58, 100), or communication breakdowns (2) did not identify the lack of mentor preparation as a contributing factor. This indicates a missed opportunity to systematize mentor development and directly link training strategies to mentorship outcomes. Moving forward, mentorship programs should prioritize deliberately designed, evidence-informed mentor training that is evaluated not only for satisfaction but also for behavioral change and outcome improvement. Our proposed framework offers a way to address this gap by grounding mentorship design in psychological, sociological, or hybrid paradigms, enabling the clear identification and definition of relevant constructs for the mentor, mentee, and the mentorship program as a whole.

### Electric mentor-mentee pairing strategies

While most studies in this scoping review utilized traditional mentor-mentee pairing approaches such as administrative assignment or faculty matching, a subset employed more eccentric and relationally attuned strategies. For example, Caine et al. (49) introduced a novel speed-dating style pairing process, allowing mentees to engage in short, structured conversations with multiple potential mentors to assess compatibility before formal selection. In a USA based study (52) a longitudinal “mentor-mentee family line” structure within a radiology residency program was introduced, where PGY-2 residents were paired with PGY-4 mentors in a near-peer format. This model created layered mentorship relationships that extended across training years, promoting sustained engagement, inter-cohort connection, and psychological safety. Residents reported enhanced comfort with clinical transitions and reduced isolation, particularly during the COVID-19 pandemic, reflecting the value of continuity-based, community-driven mentorship. Other studies (47, 57, 67) incorporated mentee-preference-based models, where personal interests, interpersonal chemistry, or mutual goals guided matching decisions. These approaches signaled a deliberate shift away from hierarchical assignment toward a more person-centered, agency-enhancing structure.

In a perspective paper by Sobel et al. (14), authors highlighted that mentor-mentee matching should consider both disciplinary alignment and training stage, particularly concerning gender-based pairing disparities. The study highlighted that women residents often reported lower satisfaction with their mentoring experiences, a trend linked to the underrepresentation of women in senior mentor roles. Although not the central focus, this observation pointed toward the importance of demographic concordance and inclusivity in matching decisions,. While Lukela et al. (84) uniquely involved peer nomination to identify potential mentors before final institutional endorsement. The intervention was specifically designed to counteract systemic gender disparities in mentorship by creating peer-based mentorship circles for women internal medicine residents. It fostered identity-affirming support structures, prioritized relational trust, and was highly rated by residents and faculty for its impact on belonging and professional development. Although these models remain under-evaluated, they demonstrate a growing recognition that initial pairing processes deeply shape the quality of mentor-mentee relationships. Future mentorship interventions should prioritize intentional, adaptive, and context-sensitive pairing methods that foster psychological safety, sustained engagement, and relational trust.

### Cultural nuances in mentorship program

While only a small number of studies in this scoping review directly addressed cultural aspects of mentorship, those that did offered important insights into how identity and inclusivity shape mentoring experiences. In a study by Engel et al. (57) where the national mentorship program in radiation oncology offered detailed insights into gender representation. The study reported that 22 mentees were female, but only six female mentors were available, leading to notable mismatches in gender concordance. An important highlight of Yehia et al. (13) is that the lack of racial/ethnic and gender-matched mentors limits mentorship effectiveness for minority groups. While the study surfaced important demographic trends, it did not incorporate validated tools for assessing cultural responsiveness or inclusivity. A study from Qatar (31) provided a particularly thoughtful example by embedding equity considerations into the evaluation design. The researchers used demographic stratified chi-square and regression analyses to explore variations in mentorship experience across cultural lines, highlighting identity’s influence in shaping perceived support and access to mentoring. However, none of the studies employed validated tools to systematically assess cultural competence, equity climate, or bias mitigation, and mainly relied instead on general self-reports and outcome comparisons. Another theoretical and non-empirical research (6) drawing upon the sociocultural mentorship models used the lens of complexity theory, emphasizing mentorship as a dynamic, evolving system shaped by institutional and interpersonal variables. Authors implicitly supported cultural responsiveness by advocating for mentorship programs to be adaptive to learners’ identities, contextual realities, and institutional structures. They advocated an institutional level, where, when aligned with strategic priorities, mentorship can facilitate gender and racial equality and improve faculty retention. However, it lacked explicit application of equity or bias mitigation tools, thereby limiting its utility for operationalizing inclusive mentorship design. In another study by Lukela et al. (84), gender disparities through a facilitated peer mentorship model for women internal medicine residents were discussed, offering identity-affirming support and fostering leadership, belonging, and professional retention. These findings highlight a critical need for future mentorship evaluations to move beyond general perception surveys and adopt validated, culturally responsive tools that can systematically assess equity, inclusion, and cultural safety thereby ensuring that mentorship programs are not only effective but also just.

### Methodological trends and future research implications

Although the majority of included studies relied on conventional methodologies and mentorship models, a few demonstrated notable innovation in either study design, evaluation approach, or application context. For example, Bassett et al. (44) employed a retrospective bibliometric analysis to correlate mentorship exposure with scholarly productivity among neurosurgery applicants. This rare but rigorous strategy introduced objective academic metrics into mentorship evaluation. While and Steinberg (28) used a multi-institutional prospective design across 96 ACGME-accredited programs, exemplifying a strategic national approach to implementation and cohort tracking, demonstrating pseudo-controlled design strengths. Another observational cohort study (51) tracked 193 surgical residents. Using structured institutional metrics and self-assessment tools, it implemented a structured, faculty-led mentorship program to improve scientific research output, including publication activity and academic participation. Findings demonstrated improvements in scholarly productivity and mentee confidence, and the model was successfully replicated across additional subspecialties, underscoring its scalability and translational value in GME mentorship design. Similarly, Ullrich, Jordan (65) used a prospective design to evaluate a structured surgical mentorship matching system contributing insight into pairing logistics and feedback mechanisms. This study implemented a matching algorithm where mentors’ strengths were intentionally leveraged to address mentees’ identified weaknesses. Residents ranked preferred mentors based on career goals and communication preferences, and structured feedback cycles were embedded into the pairing process. Results showed that 92% of residents were satisfied with their mentorship experience, with gains reported in career guidance, emotional support, and mentor accessibility. Another study worth mentioning, which deserves attention due to its methodological section, is the pre-post intervention design, which evaluated mentorship outcomes in a trauma and emergency surgery context using both mentor and mentee perspectives (59). These examples underscore the potential for greater methodological creativity and contextual adaptability in mentorship research and highlight models worth building upon in future studies.

### Strengths and limitations

To our knowledge, this scoping review represents the most comprehensive synthesis of mentorship interventions within Graduate Medical Education (GME) to date. By analyzing 94 studies spanning multiple disciplines, stages of training, and global contexts, we identified key structural elements, theoretical frameworks, evaluation approaches, and outcome domains that characterize mentorship programs in GME. This breadth allowed us to map the field systematically and derive insights relevant to both research and practice.

However, several limitations warrant consideration. This scoping review was limited to four major databases—PubMed, Scopus, CINAHL, and Embase—which, while extensive, may not have captured the full scope of literature available in discipline-specific or regional repositories. This exclusion may have introduced selection bias and affected the comprehensiveness of this scoping review. Additionally, although all stages of the review process study selection, data extraction, and synthesis were independently verified by multiple researchers to ensure methodological rigor, the interpretive nature of qualitative synthesis means that a degree of subjectivity cannot be fully eliminated. Nonetheless, transparent documentation, collaborative consensus, and adherence to scoping review guidelines were employed to mitigate these risks.

## Conclusion

In conclusion, this scoping review highlights several critical gaps and recurring patterns within the GME mentorship literature. Key themes include the inconsistent application of theoretical models, underutilization of validated evaluation tools, limited training and support for mentors, and an over-reliance on traditional one-on-one faculty-trainee models, often at the expense of more inclusive formats such as peer, near-peer, or group mentorship. There is a clear and urgent need for mentorship interventions that are context-sensitive, developmentally appropriate, and conceptually grounded. Future work should prioritize strengthening the mentorship evidence base, which will require both empirical rigor and strategic alignment with institutional goals related to cultural, leadership, well-being, and academic success. The conceptual framework developed through this scoping review offers a practical tool for educators, researchers, and institutional leaders to design, implement, and evaluate evidence-informed and outcomes-driven mentorship interventions. When applied intentionally, this framework can potentially support the creation of robust, sustainable, and high-impact mentorship cultures across GME settings worldwide.

## Data Availability

The original contributions presented in the study are included in the article/Supplementary material, further inquiries can be directed to the corresponding author.
