# Supplementary material for: A scoping review of mentorship in Graduate Medical Education: a proposed conceptual framework
Source: Front Med (Lausanne). 2025 Aug 18;12:1616148. doi: 10.3389/fmed.2025.1616148 (PMC12400154; doi:10.3389/fmed.2025.1616148)
Supplement: Supplementary file 1 [file Table_1.docx]

**Appendix I:** **Search Strategy for All Databases**

**PubMed**

Date of search: 19 February 2025
Records retrieved: 1,697
Search string:

(("education, medical, graduate"[MeSH Terms] OR ("education"[All Fields] AND "medical"[All Fields] AND "graduate"[All Fields]) OR "graduate medical education"[All Fields])

AND ("mentors"[MeSH Terms] OR "mentors"[All Fields] OR "mentorship"[All Fields] OR "mentorships"[All Fields]))

AND (2015:2025[pdat])

**Scopus**

Date of search: 19 February 2025
Records retrieved: [144]
Search string:

TITLE-ABS-KEY("graduate medical education" OR "residency training" OR "postgraduate medical education")

AND TITLE-ABS-KEY("mentorship" OR "mentor*" OR "mentoring")

AND PUBYEAR > 2014 AND PUBYEAR < 2026

**CINAHL (EBSCOhost)**

Date of search: 19 February 2025
Records retrieved: [272]
Search string:

(MH "Education, Medical, Graduate") OR (TX "graduate medical education" OR "residency training")

AND (TX "mentorship" OR "mentoring" OR "mentors")

AND (PY 2015-2025)

**Embase (via Elsevier)**

Date of search: 19 February 2025
Records retrieved: [222]
Search string:

('graduate medical education'/exp OR 'residency training'/exp OR 'postgraduate medical education')

AND ('mentorship'/exp OR mentorship:ti,ab,kw OR mentoring:ti,ab,kw OR mentor*:ti,ab,kw)

AND [2015-2025]/py

**Note:** Search strings were adapted to each database’s indexing structure and controlled vocabulary (e.g., MeSH in PubMed, EMTREE in Embase, CINAHL Subject Headings). No language filters were applied. Forward/backward citation tracking and grey literature (n=30) were also reviewed.
